# ABCG1 promotes the proliferation and migration of clear cell renal cell carcinoma and reduces its apoptosis

**DOI:** 10.7150/ijms.107055

**Published:** 2025-05-30

**Authors:** Yihan Dong, Qiufang Qiao, Shaomin Guo, Ruibing Chen, Tianyu Lin, Xinyu Liu, Jiaxin Li, Shiming Liu, Huamao Jiang, Yong Wang, Dan Yue, Rui Wang

**Affiliations:** 1 School of Medical Technology, Tianjin Medical University, Tianjin 300203, China; 2 Department of Blood Transfusion, Cangzhou Hospital of Integrated Traditional and Western Medicine, Cangzhou 061012, China; 3 School of Pharmaceutical Science and Technology, Faculty of Medicine, Tianjin University, Tianjin 300072, China; 4 Department of Urology, Tianjin Institute of Urology, The second Hospital of Tianjin Medical University, Tianjin Medical University, Tianjin 300211, China; 5 Tianjin Human Sperm Bank, Tianjin Institute of Urology, The Second Hospital of Tianjin Medical University, Tianjin 300211, China; 6 Department of Urology, The First Affiliated Hospital of Jinzhou Medical University, Jinzhou, Liaoning 121000, China

**Keywords:** Clear cell renal cell carcinoma, ABCG1, Benzamil

## Abstract

**Background and Aims:** In this study, we analyzed the expression levels of ATP-binding cassette transporter G1 (ABCG1) in clear cell renal cell carcinoma (ccRCC) to assess its significance for early diagnosis and its role in the disease's biological processes.

**Materials and Methods:** ABCG1 expression levels in ccRCC were determined by analyzing The Cancer Genome Atlas (TCGA) database, as well as immunohistochemical staining and western blot. The correlation between the ABCG1 expression level and the clinicopathological stage and diagnostic prognosis of ccRCC were analyzed by the TCGA database. The ABCG1 stable knockout cell line and functional experiments using ABCG1 inhibitors were constructed to verify the effect of reduced ABCG1 expression on the ccRCC cell function. In addition, the pathways and genes affected by ABCG1 were analyzed by RNA-seq.

**Results:** Analysis of the TCGA database demonstrated, as well as immunohistochemical staining and western blot detection, confirmed that ABCG1 was significantly elevated in ccRCC. The expression level of ABCG1 was correlated with the clinicopathological stage of ccRCC. The Cox regression analysis showed that ABCG1 could be used as an independent prognostic marker for ccRCC. And decreasing ABCG1 expression decreased the proliferation, migration, and invasion abilities of ccRCC cells, with increased apoptosis. In addition, analysis of ABCG1-related differentially expressed genes (DEGs) showed that ABCG1 positively regulates specific proliferation-related genes and negatively affects those associated with apoptosis.

**Conclusions:** In summary, these findings highlight the critical role of ABCG1 in ccRCC progression and suggest its potential as a biomarker for diagnosis and prognosis.

## Introduction

Renal cell carcinoma (RCC) represents 2% to 3% of all cancer cases, with three principal histologic subtypes: clear cell renal cell carcinoma (ccRCC), papillary carcinoma, and chromophobe carcinoma 1. Global statistics from 2020 indicate approximately 431,288 new RCC cases and 179,358 related deaths annually 2, ranking it as the 13th leading cause of cancer 3. RCC is the sixth most prevalent cancer among men and the tenth most common among women, exhibiting higher morbidity and mortality rates in males 2. The incidence of kidney cancer has shown a yearly increase and varies significantly across different regions, with rates in Europe and North America being notably higher than in Asia, as well as being more prevalent in developed countries compared to developing nations. In China, the crude incidence rate of kidney cancer was reported at 54.8% in 2016, with a crude death rate of 19.5%. Furthermore, the age-standardized incidence rate of kidney cancer was higher in urban areas (41%) than in rural areas (25%) 4.

The precise mechanisms underlying RCC pathogenesis remain poorly understood. Studies have identified risk factors like smoking, obesity, hypertension, and diabetes mellitus, along with lifestyle choices and environmental exposures 5. Traditional treatments, such as laparoscopic surgery, have limitations in controlling tumor growth and spread, focusing on extending survival and improving quality of life. Consequently, early diagnosis of RCC is paramount for optimal patient management and mortality reduction. However, a definitive screening modality and approach remain to be established, emphasizing the need for improved prognostic assessment and treatment selection for RCC patients 6.

Tumor markers are proteins or other molecules that can be detected in body fluids, including those present in tumor tissues or blood, such as cell surface antigens, metalloproteinases, proteins, and nucleic acids. Consequently, the detection of tumor markers holds significant importance for assessing tumor occurrence, progression, and prognosis. Urine is a commonly used non-invasive bodily fluid in clinical settings due to its easy collection and large volume, making it an ideal candidate for developing non-invasive renal function tests. Urinary protein content is abundant and stable, allowing for non-invasive detection, and presents potential as a biomarker for the early diagnosis and prognosis of kidney cancer. It also shows promise for targeted kidney cancer therapy, highlighting the need for further research to enhance its clinical application.

The ATP-binding cassette (ABC) family of transporters represents a class of transmembrane proteins that use ATP to facilitate energy-dependent processes. Humans have 48 classes of ABC transporters grouped into seven subfamilies (ABCA to ABCG) 7. Structurally, ABC transporters can be divided into two principal categories: complete transporters comprising two hemicarriers (e.g., ABCA) and a hemicarriers (e.g., ABCG), typically functioning as dimers 8. ABCG1 plays a pivotal role in the efflux of intracellular cholesterol. Prior studies have indicated a correlation between serum cholesterol levels and ccRCC invasion and prognosis, suggesting that cholesterol metabolism may influence ccRCC metastasis 9. The function of the ABCG1 is predominantly associated with cellular cholesterol efflux, highlighting its potential relevance in ccRCC tumorigenesis and progression. Furthermore, ABCG1 can modulate the tumor microenvironment by regulating macrophage activity, thereby promoting tumor growth. Recent research suggests ABCG1 may serve as a promising biomarker for renal cell carcinoma. For instance, Meng 10 demonstrated that ABCG1 could act as a prognostic and diagnostic biomarker for ccRCC. However, this study's observational nature imposes limitations, thereby necessitating further validation. Additionally, Li 11 suggested that ABCG1 may represent a viable therapeutic target for RCC.

Our study evaluated the expression and diagnostic relevance of ABCG1 in ccRCC by analyzing data from a public online database. The results confirmed high ABCG1 expression in ccRCC, consistent with clinical samples. The knockout of ABCG1 was observed to inhibit both the proliferation and migration of ccRCC cells while simultaneously increasing apoptosis. RNA-sequencing was then used to explore the underlying mechanisms. Furthermore, for the first time, the ABCG1 inhibitor Benzamil was introduced in a ccRCC cell line, indicating that ABCG1 may play a crucial role in the etiology and progression of renal cancer. These findings could enhance the optimization of diagnosis and treatment strategies for ccRCC patients.

## Materials and Methods

### Expression analysis of ABCG1 in ccRCC

The Cancer Genome Atlas (TCGA) website (https://portal.gdc.cancer.gov/) serves as an interactive portal 12. In this study, we downloaded and compiled RNA sequencing data from the STAR process across 33 tumor projects within the TCGA database, extracting data in TPM format. We analyzed the mRNA expression levels of the ABCG1 gene in 541 clear cell renal cell carcinoma (KIRC) tissues alongside 72 adjacent non-tumor tissues, investigating the relationship between ABCG1 expression and the clinicopathological features, diagnosis, and prognosis of renal cancer. The Mann-Whitney U test was employed for unpaired samples, while paired samples were evaluated using the paired samples t-test. Additionally, UALCAN is another interactive portal (https://ualcan.path.uab.edu/) that facilitates comprehensive analysis of databases like TCGA, MET500, CBTTC, and CPTAC 13. In this study, we utilized UALCAN to examine the protein expression levels of ABCG1 in ccRCC tissues and healthy human kidney tissues, utilizing data provided by the CPTAC and ICPC databases.

### Immunohistochemistry (IHC)

In this study, cancer tissues and adjacent samples were obtained from 68 patients diagnosed with ccRCC at the Second Hospital of Tianjin Medical University. Data collected included hospitalization numbers, gender, age, tumor size, TNM stage, and Fuhrman grade. This research received approval from the institutional ethics committee, and informed consent was obtained from all patients. The tissues were sectioned at a thickness of 2 μm, followed by deparaffinization and hydration. Antigen retrieval was performed by heating in a 98 ºC water bath using 0.01 M sodium citrate buffer (pH 6.0). Enzymatic activity was blocked with 3% H_2_O_2_ at room temperature in the dark for 15 min, and then samples were incubated with a 5% BSA solution for one hour. The primary antibody for ABCG1 (1:50; ABclonal, China) was applied overnight at 4 ºC, followed by incubation with a secondary antibody (ZSGB-BIO, China) for one hour at room temperature. Color development was achieved using an HRP DAB kit (ZSGB-BIO, China), and hematoxylin counterstaining was performed before mounting the samples. Staining intensity was scored as follows: 0 (colorless), 1 (tawny), and 2 (tan). The proportion of positive cells was categorized as: 0 (0 - 1%), 1 (1% - 5%), 2 (6% - 10%), 3 (11% - 20%), 4 (21% - 50%), and 5 (> 50%). The final score was derived from the sum of staining intensity and the proportion of positive cells, with scores < 4 indicating negative results and ≥ 4 indicating positive results.

### Collection and processing of urine samples

The urine samples required for this study were provided through the Clinical Samples and Data Resource Center of the Second Hospital of Tianjin Medical University. The morning urine of 7 patients with renal cell carcinoma and 6 healthy people admitted to the Second Hospital of Tianjin Medical University were collected, and 7 patients had T1-T4 stage without metastasis, 3 males and 4 females. Urine from kidney cancer patients and healthy patients was collected in the middle of the morning, centrifuged at 2000×g at 4℃ for 20 min, and the precipitate was discarded in a new tube. 400 μL of urine was added to the inner tube of the cleaned ultrafiltration tube, centrifuged at 12000×g at 4℃ for 30 min, repeated 5 times, until 2 mL was filtered. After 2 washes with PBS, the liquid in the ultrafiltration tube was transferred to a 1.5 mL centrifuge tube and stored in a -80 ºC ultra-low temperature freezer for subsequent experiments.

### Western blot (WB)

Cell and tissue protein extracts were prepared using a 10:1 ratio of SDS protein lysate to a 10× cocktail. Protein concentration was assessed with the BCA protein assay kit (Solarbio, China), utilizing a standard curve generated from bovine serum albumin (BSA). SDS-PAGE gels (10% separating gel and stacking gel) were constructed according to protein molecular weights. Electrophoresis was conducted at 80 V, increasing to 120 V once the samples exited the separating gel and marker bands were resolved. The transfer was performed at 250 mA for 120 min using PVDF membrane (Millipore, USA). Afterward, membranes were blocked with 5% skimmed milk powder at room temperature for 2 h. The ABCG1 antibody (1:2000; ABclonal, China) and β-actin antibody (1:3000; Affinity, USA) were incubated overnight at 4 ºC. Subsequently, HRP-labeled goat anti-rabbit/mouse IgG antibody (1:3000; Affinity, USA) was applied for one hour at room temperature. Detection was performed using ECL chemiluminescence solution (Millipore, Germany) under a chemiluminescence imaging system.

### Prognosis and clinicopathological characteristics of ABCG1 in ccRCC

The clinical data of 541 patients with ccRCC from the TCGA database were analyzed, focusing on pathological stage, T stage, N stage, and M stage. The receiver operating characteristic curve (ROC) was employed to assess the diagnostic value of ABCG1 in ccRCC; an area under the curve (AUC) value closer to 1.0 indicates a higher diagnostic accuracy. Additionally, the Kaplan-Meier plotter (www.kmplot.com) was utilized to examine the association between ABCG1 expression and prognosis in ccRCC patients. Both univariate and multivariate Cox regression analyses were conducted to evaluate the prognostic significance of ABCG1 expression alongside other clinicopathological factors. Among them, 541 ccRCC were divided into ABCG1 high expression group (n = 271) and low expression group (n = 270) according to the median ABCG1 expression.

### Cell culture

All the cell lines used were purchased from the American Type Culture Collection Library (ATCC). Complete medium was prepared with 45 mL of MEM/DMEM medium (Viva Cell, Germany), 5 mL of fetal bovine serum (Viva Cell, Germany) and 0.5 mL of Penicillin-Streptomycin solution (Viva Cell, Germany), and 5 cell lines HEK-293T, ACHN, A498, 786-O and HK-2 were cultured in a cell culture incubator (37 °C, 5% CO_2_).

### Construction of the plasmids and the stable cell lines

The sgRNA of *ABCG1* (F: 5'-CACCGATTATGGGTCCTTCCGGGGC-3', R: 5'-AAACGCCCCGGAAGGACCCATAATC-3') was designed via the CRISPOR website (http://crispor.tefor.net/). The sgRNA was ligated into the lentiCRISPRv2 plasmid. After transformation, amplification and extraction, empty plasmid (lentiCRISPRv2) and ABCG1-KO plasmids were transfected into ACHN, A498 and 786-O cell lines by lentiviral infection, and after puromycin selection, gene knockout efficiency was confirmed by Western blot and RT-qPCR.

### Real-time RT-PCR (RT-qPCR)

The upper RNA was absorbed by adding Trizol and DNA extract (DNA extract: Trizol = 1:5). After adding isopropanol and washing with 75% ethanol, RNA was obtained by resuspension in sterile enzyme-free water and reverse transcribed to yield cDNA. The cDNA, primer, 2× ChamQ Universal SYBR qPCR Master Mix (Vazyme Biotech, China) were configured in the corresponding proportion, and the reaction procedure was set: 95 ºC predenaturation for 1min; 95 ºC 10 s, 60 ºC 30 s, denaturation, annealing and extension for 40 cycles; 95 ºC 30 s, 60 ºC 30 s, 95 ºC 30 s. The expression of the target genes was normalized by GAPDH and analyzed by the 2^-ΔΔCt^ method. The primer sequences were as follows:

*GAPDH* (F: 5'-TGCACCACCAACTGCTTAGC-3', R: 5'-GGCATGGACTGTGGTCATGAG-3');

*ABCG1* (F: 5'-GTCCTCTTTCCCGCATTATCTGG-3', R: 5'-AGTTCCTGGAAGGTCTTGTTCAC-3');

*TP53* (F: 5'-CCTCAGCATCTTATCCGAGTGG-3', R: 5'-TGGATGGTGGTACAGTCAGAGC-3');

*CDKN1A* (F: 5'-AGGTGGACCTGGAGACTCTCAG-3', R: 5'-TCCTCTTGGAGAAGATCAGCCG-3');

*BBC3* (F: 5'-ACGACCTCAACGCACAGTACGA-3', R: 5'-CCTAATTGGGCTCCATCTCGGG-3');

*BMF* (F: 5'-CAGTGGCAACATCAAGCAGAGG-3', R: 5'-GCAAGGTTGTGCAGGAAGAGGA-3');

*BCL2L15* (F: 5'-ATTGCTGGTCGCCTTCGGATGT-3', R: 5'-CCACAGTGTCCTGGAGTATAGC-3');

*FGF5* (F: 5'-GGAATACGAGGAGTTTTCAGCAAC-3', R: 5'-CTCCCTGAACTTGCAGTCATCTG-3');

*PGF* (F: 5'-GGCGATGAGAATCTGCACTGTG-3', R: 5'-ATTCGCAGCGAACGTGCTGAGA-3').

### Colony formation assay

For colony formation assays, cells were seeded at a density of 500 cells per well in a 6-well plate and gently shaken to ensure even distribution. Cultures were maintained for 10 days, with media replenished every three days. After fixation with 4% paraformaldehyde and staining with crystal violet, images were captured, and colony areas were quantified using ImageJ software.

### Cell proliferation assay

Cells were plated at a density of 1000 cells per well in 96-well plates. Following incubation periods of 0, 24, 48, 72, and 96 h, 10 μL of CCK-8 reagent (Biosharp, China) was added to each well. The plates were then incubated at 37℃ for 2 h in the dark, after which the absorbance was measured at a wavelength of 450 nm.

### Cell migration and invasion assays

In the migration experiment, 600 μL of basal medium with 20% FBS was added to each well, China 200 μL of ACHN and A498 cell suspension containing MEM minimal medium was added to each well, 15×10^4^ ACHN cells per chamber, 6×10^4^ cells per chamber for A498 cells. In the invasion experiment, Matrigel (BD, USA) (50 μL/chamber) was evenly plated in a transwell chamber for 2 h in a 37 °C incubator before cells were added, and the subsequent steps were the same as for the migration experiment. Observe every 3 h for cells to pass through the lower chamber, and after 10 h, there are cells to pass through. After fixation with 4% paraformaldehyde, stain with Giemsa and wash in PBS, wipe off the inner uncrossed cells. 5 fields of view were randomly selected at 200× field to count the number of cells.

### Apoptosis experiments

For cell analysis, EDTA-free Trypsin was utilized for cell digestion, and the resulting cell suspension was transferred to a 1.5 mL tube. After centrifugation, the supernatant was discarded, and the cells were resuspended in PBS. This process was repeated once more before adding 300 μL of 1× Binding Buffer. The mixture was gently pipetted to ensure homogenization, followed by the addition of 5 μL of Annexin V-FITC. After thorough mixing, the sample was incubated in the dark for 15 min. Subsequently, 5 μL of PI solution was incorporated, mixed again, and analyzed using a flow cytometer.

### Structural characterization of ABCG1 and molecular docking with drugs

The AlphaFold protein structure database (https://alphafold.ebi.ac.uk/) serves as a widely accessible resource for high-precision protein structure predictions 14. Additionally, the PDB Protein Data Bank (https://www.rcsb.org/pdb/) is recognized as the sole global repository for structural data on biological macromolecules 15. In this investigation, ABCG1 was sourced from PDB identification number 7R8E, while the ABCG1 monomer was obtained via AlphaFold identification number AF-P45844-F1. The molecular structure of Benzamil was retrieved from the PubChem database (https://pubchem.ncbi.nlm.nih.gov/). Utilizing the CB-Dock2 database (https://cadd.labshare.cn/cb-dock2/php/blinddock.php) 16, three potential docking outcomes were generated by submitting the structures of Benzamil and ABCG1. All structural diagrams were rendered using PyMOL.

### Cytotoxicity assay

Cells are seeded at a density of 5,000 cells per well on 96-well plates. After the cells were completely adherent, the medium was discarded and medium prepared with Benzamil hydrochloride (MedChemexpress, United States) was added at concentrations of 1, 5, 10, 20, 40, and 80 μM, and placed in an incubator for 24 h. 10 μL of CCK-8 reagent (Biosharp, China) was added to each well, incubated at 37 °C for 2 h in the dark, and the absorbance was measured at 450 nm, and the drug inhibition curve of the cells was calculated. Subsequent cell phenotyping experiments were performed with a drug concentration of 10 μM in the experimental group, and the same concentration of DMSO was added to the control wells.

### Transcriptome sequencing

When the cells grew to 80-90%, the medium was discarded, 1 mL of Trizol was added and pipetted repeatedly, collected into a 1.5 mL enzyme-free EP tube, and stored in a -80 °C freezer after liquid nitrogen quick-freezing. Three generations of cell RNA were collected and sent to Shanghai Sangon Bioengineering Co., Ltd. for gene expression profiling analysis through the gene chip platform to identify differential genes.

### Statistical method

Data processing was performed using IBM SPSS Statistics 25.0 and GraphPad Prism 9.0 software and statistics were presented in mean ± SEM form, with paired t-test, Mann-Whitney U test, chi-square test, Spearman correlation coefficient analysis, univariate COX regression model selected according to the different experimental samples. * *P* < 0.05 was considered to be statistically significant. ns, *P ≥* 0.05, *, *P* < 0.05, **, *P* < 0.01, ***, *P* < 0.001, ****, *P* < 0.0001.

## Results

### ABCG1 is highly expressed in ccRCC

**Figure 1:**
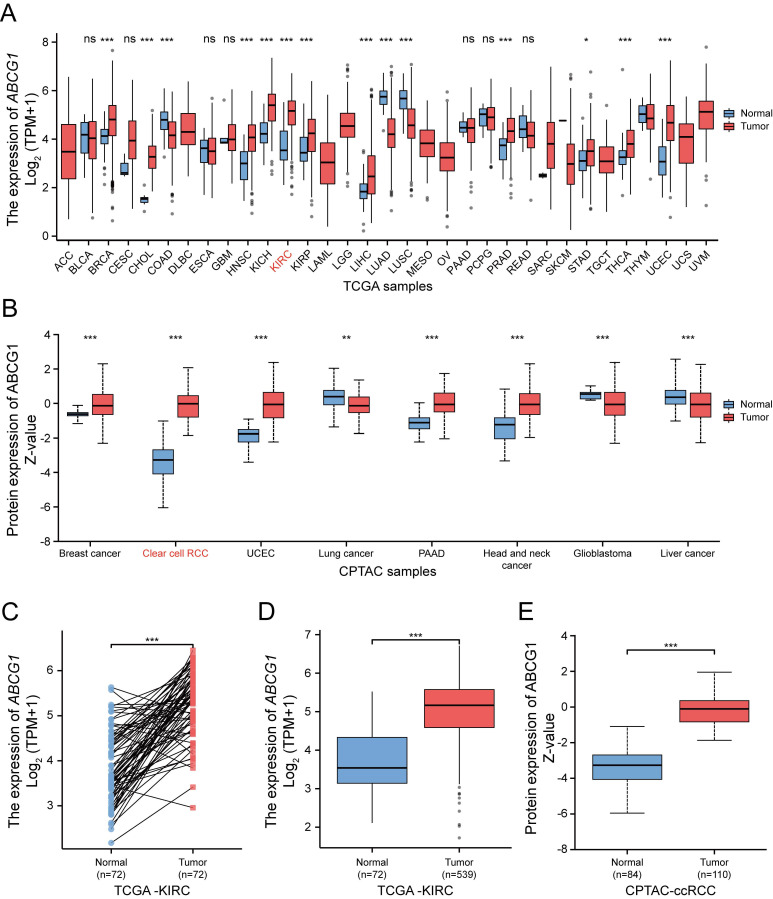
Bioinformatic analysis of the expression of ABCG1 in ccRCC. **A** TCGA database of *ABCG1* mRNA levels in 33 different tumor types. **B** Protein levels of ABCG1 in 8 different tumor types in the CPTAC database. **C, D** Paired (**C**) and unpaired (**D**) in the TCGA database were used to analyze the mRNA levels of *ABCG1* in ccRCC and normal kidney tissues. **E** Protein levels of ABCG1 in ccRCC and normal renal tissue in the CPTAC database. ns, *P* ≥ 0.05, *P < 0.05, **P < 0.01, ***P < 0.001.

The transcriptional expression of *ABCG1* across 33 different tumor types and adjacent tissues (n = 10,534) was assessed via RNA sequencing data extracted from the TCGA database (Figure 1A). Our findings revealed that *ABCG1* expression was elevated in the majority of cancer tissues, including ccRCC, in comparison to paracancerous tissues. Protein expression levels of ABCG1 in a pan-cancer context were examined using the UALCAN cancer database, disclosing that ABCG1 levels in ccRCC (n = 110) were significantly higher compared to those in normal tissues (n = 84) (Figure 1B). Further analysis, both on paired (Figure 1C) and unpaired (Figure 1D) samples, corroborated the increase in mRNA expression of *ABCG1* in renal cancer tissues (n = 539) relative to adjacent tissues (n = 72), with correspondding protein expression levels also elevated (Figure 1E). Preliminary confirmation through database analysis indicated that both mRNA and protein levels of ABCG1 are significantly heightened in ccRCC.

**Figure 2:**
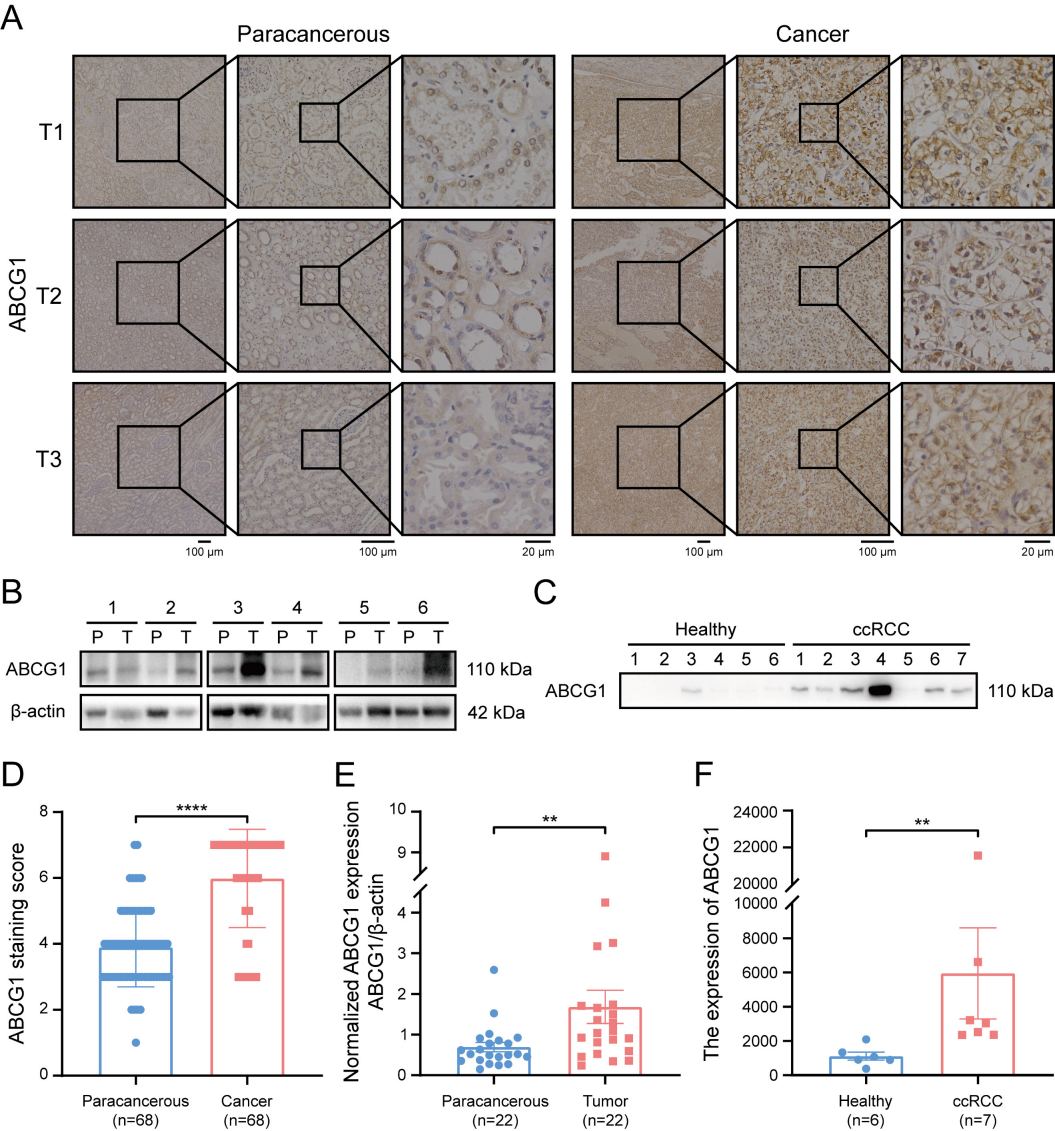
Expression of ABCG1 in tissues and urine of ccRCC patients. **A** Immunohistochemistry staining detected the expression of ABCG1 in cancer and adjacent tissues of 68 ccRCC patients. **B** Western blot detects the expression of ABCG1 in cancer and para-cancerous tissues of 22 ccRCC patients (T: tumor tissue, P: corresponding para-cancerous tissue). **C** Western blot was used to detect the expression of ABCG1 in the urine of 7 ccRCC patients and 6 healthy people. After quantification of BCA protein, equal amounts of protein were loaded. **D** Statistical chart of ABCG1 staining scores in ccRCC and adjacent tissues. **E** Use Image J software to quantitatively analyze ABCG1 in ccRCC and adjacent tissues. **F** Image J software was used to quantitatively analyze ABCG1 in the urine of ccRCC patients and healthy people. **P < 0.01, ****P < 0.0001.

Subsequently, immunohistochemical staining was performed on 68 pairs of ccRCC and adjacent tissue sections (Figure 2A). Protein extraction from 22 matched pairs of cancerous and correspondingly para-cancerous tissues further substantiated our findings (Figure 2B). Additionally, morning urine samples were procured from 7 ccRCC patients and 6 healthy individuals (Figure 2C). Following ultrafiltration for urinary protein extraction and subsequent calibration via the BCA method. Our results demonstrated a marked increase in ABCG1 protein levels in ccRCC tissues when compared to adjacent tissues (*P* < 0.0001) (Figure 2D). Statistical analysis of immunohistochemical staining scores indicated no significant correlation between ABCG1 expression levels and patient factors such as age, gender, tumor size, T stage, or Fuhrman grade (Table 1). Furthermore, western blot results corroborated the significant elevation of ABCG1 expression in ccRCC tissues compared to para-cancerous tissues (*P* < 0.01) (Figure 2E). Urinary analysis corroborated these findings, revealing increased *ABCG1* expression in the urine of ccRCC patients compared to healthy controls (*P* < 0.01) (Figure 2F). Collectively, these experiments established ABCG1 is highly expressed in ccRCC, demonstrating its potential as a non-invasive biomarker for the diagnosis of ccRCC.

### *ABCG1* is associated with clinicopathological characteristics and diagnosis and prognosis of ccRCC patients

**Figure 3:**
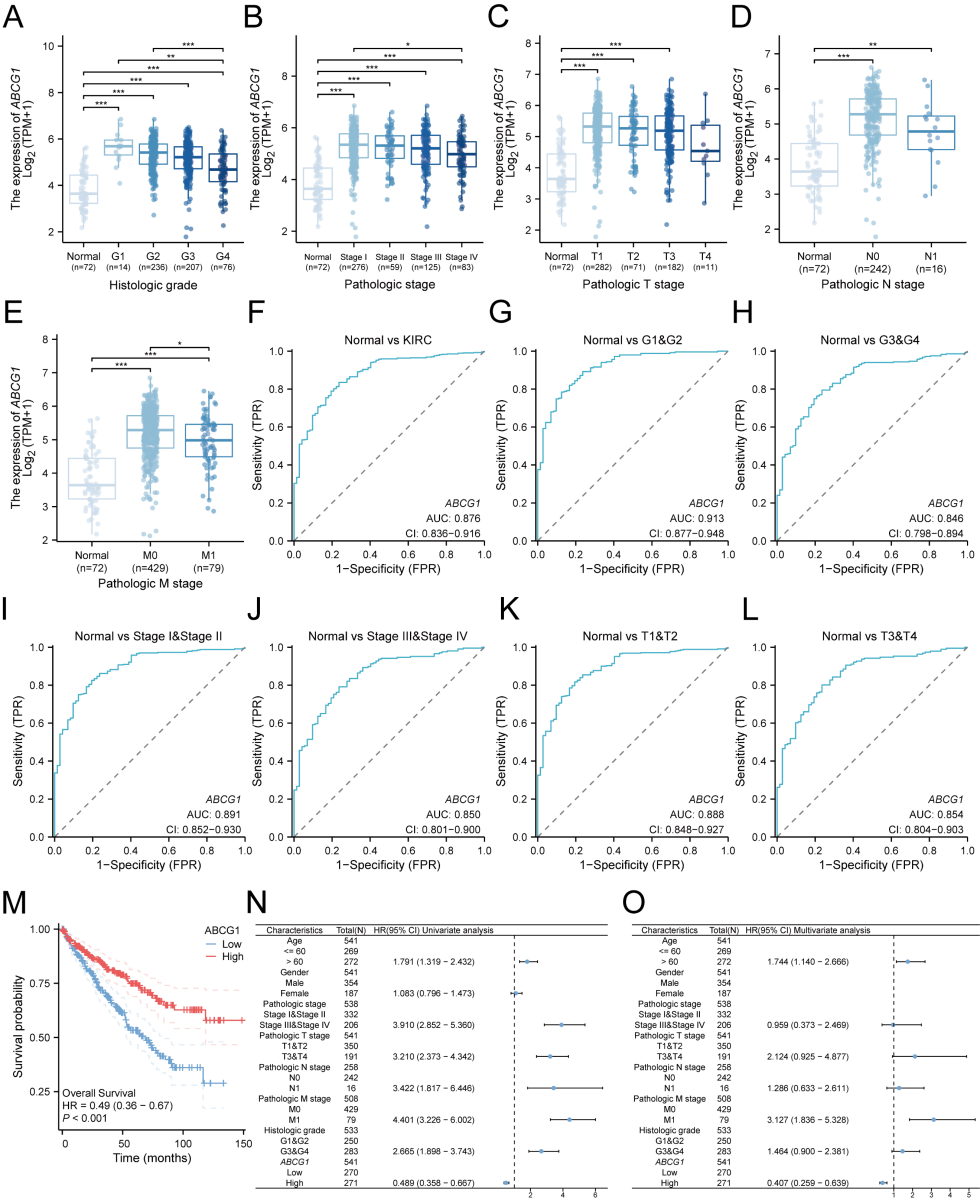
Association between *ABCG1* expression and clinicopathological features and diagnostic prognosis of ccRCC in the TCGA database.** A-E** Association between *ABCG*1 expression and the clinicopathological features of ccRCC, (**A**) histological grade, (**B**) pathological grade, (**C**) T stage, (**D**) N stage, and (**E**) M stage. **F-L** ROC curve of *ABCG1* for identifying normal tissue and renal cancer tissue with clinicopathological features. (**G-H**) different histological grades, (**I-J**) different pathological grades, and (**K-L**) different T stages. **M** Association between *ABCG1* expression and patient OSt in ccRCC patients. **N-O** univariate (**N**) and multivariate (**O**) Cox regression forest plot. *P < 0.05, **P < 0.01, ***P < 0.001.

We subsequently investigated the relationship between *ABCG1* gene expression and various clinicopathological parameters among ccRCC patients. Our analysis utilized clinical data sourced from 541 ccRCC patients available in the TCGA database, which included information on their histological grade, pathological grade, T stage, N stage, and M stage. Our results indicated a significant association between *ABCG1* expression levels and the histological grade (Figure 3A), pathological stage (Figure 3B), T stage (Figure 3C), N stage (Figure 3D), and M stage (Figure 3E) of ccRCC patients. Specifcally, diminished *ABCG1* expression was observed in advanced stages and high-grade differentiated ccRCC.

Receiver Operating Characteristic (ROC) analysis was employed to evaluate the diagnostic accuracy of* ABCG1* in ccRCC, yielding a noteworthy area under the curve (AUC) of 0.876 (Figure 3F). Furthermore, *ABCG1* exhibited differential diagnostic capability for ccRCC, in which the AUC for G1 & G2 (AUC = 0.913) surpassed that for G3 & G4 (AUC = 0.846) (Figure 3G-H). Similarly, AUC values for Stage I & Stage II (AUC = 0.891) outperformed Stage III & Stage IV (AUC = 0.850) (Figure 3I-J), and T1 & T2 (AUC = 0.888) were greater than T3 & T4 (AUC = 0.854) (Figure 3K-L). These findings suggest that *ABCG1* presents enhanced diagnostic capabilities for ccRCC patients exhibiting lower stage grades.

Additionally, we noted a significant relationship between *ABCG1* expression and overall survival (OS) (HR = 0.49, *P* < 0.001) (Figure 3M), low *ABCG1* gene expression has a poor prognosis. Hence, we delved deeper into the prognostic implications of *ABCG1* in ccRCC, which were further elucidated through univariate and multivariate Cox regression analyses conducted on TCGA data (Table 2). Our analyses indicated that age (*P* = 0.010), M stage (*P* < 0.001), and *ABCG1* (*P* < 0.001) were established as independent prognostic factors for OS in ccRCC patients (Figure 3N, O).

### ABCG1 is associated with the proliferation, migration, invasion and apoptosis of ccRCC cells

**Figure 4:**
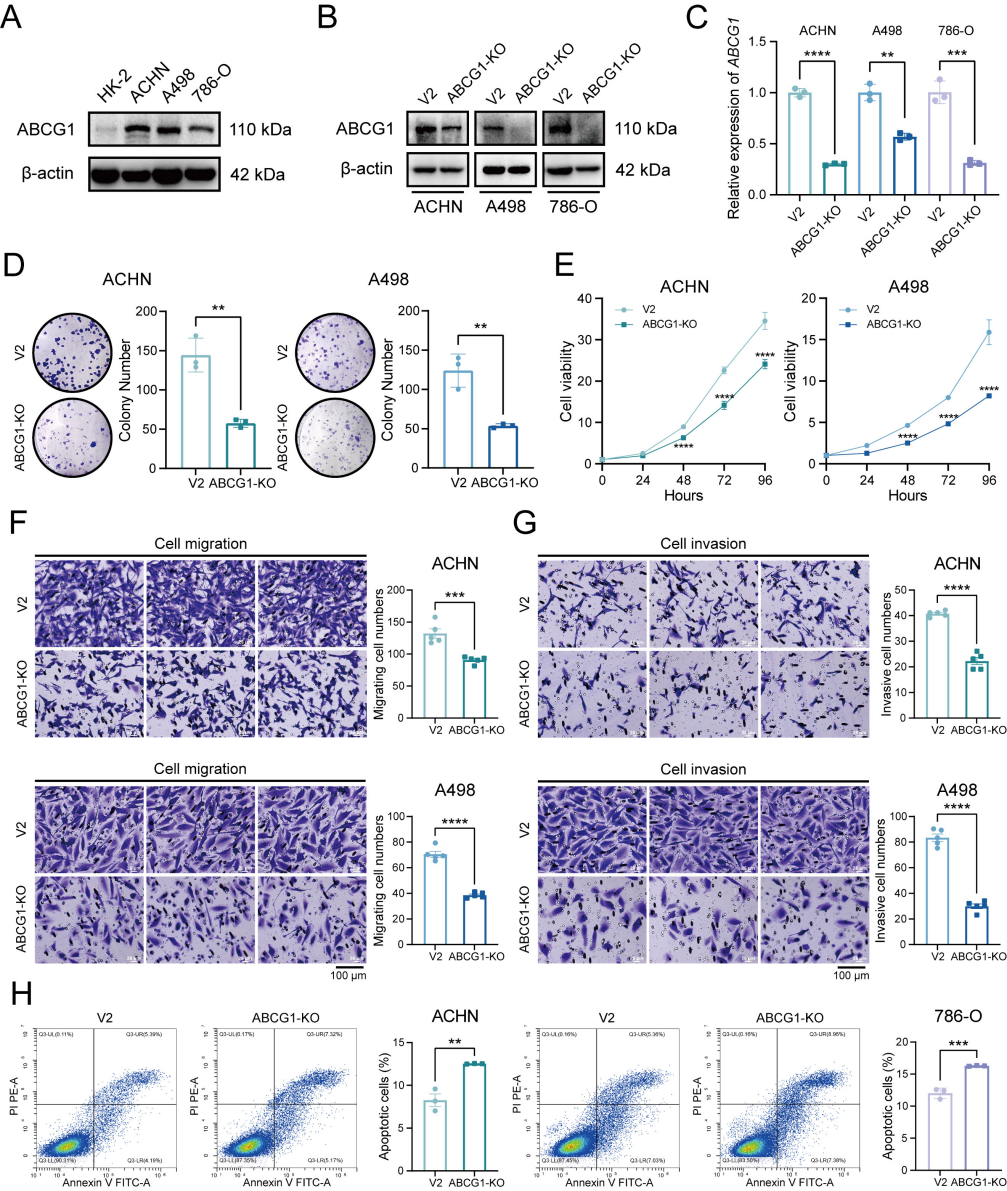
Effect of ABCG1 knockout on the function of ccRCC cells. **A** Western blot was used to detect the expression level of ABCG1 protein in ccRCC cell lines HK-2, ACHN, A498 and 786-O. **B** Western blot was used to detect the expression level of ABCG1 protein in renal cancer cell lines of V2 control group and ABCG1-KO group. **C** RT-qPCR was used to detect the expression level of *ABCG1* mRNA in renal cancer cell lines of V2 control group and ABCG1-KO group. **D** Plate clone formation assay of V2 control group and ABCG1-KO group in ACHN and A498 cell lines. **E** CCK-8 proliferation assay of V2 control group and ABCG1-KO group in ACHN and A498 cell lines. **F-G** Migration (**F**) and invasion (**G**) assay of V2 control group and ABCG1-KO group in ACHN and A498 cell lines. **H** Flow cytometry was used to detect the percentage of apoptosis of V2 control group and ABCG1-KO group in ACHN and 786-O cells. **P < 0.01, ***P < 0.001, ****P < 0.0001.

Given the elevated expression of ABCG1 in renal cancer cell lines, including ACHN, A498, and 786-O (Figure 4A), We proceeded to construct ABCG1 knockout (hereinafter referred to as KO) cell lines and the corresponding control (hereinafter referred to as V2) cell lines to further explore the effects of ABCG1 deletion on the biological functions of ccRCC cells. The verification of these cell lines was conducted through western blot (Figure 4B) and RT-qPCR (Figure 4C) assays, confirming a decrease in ABCG1 levels in the knockout lines. Clonogenic and CCK-8 proliferation assays indicated that the knockout of ABCG1 resulted in diminished colony size and count, as well as a reduction in growth rate, thereby suggesting an inhibitory effect on the proliferative capacity of ccRCC cells (Figure 4D, E). Subsequently, transwell assays demonstrated that ABCG1 knockout led to a reduction in the number of cells traversing the chamber, indicating an impairment in both the migratory and invasive abilities of ccRCC cells (Figure 4F, G). Concurrently, cell apoptosis was assessed via flow cytometry, revealing an increase in apoptotic levels following ABCG1 knockout (Figure 4H). Collectively, these findings suggest that ABCG1 knockout may diminish the proliferation, migration, and invasion capabilities of ccRCC cells *in vitro* while simultaneously promoting apoptosis.

### The ABCG1 inhibitor Benzamil has been implicated in ccRCC cell function

**Figure 5:**
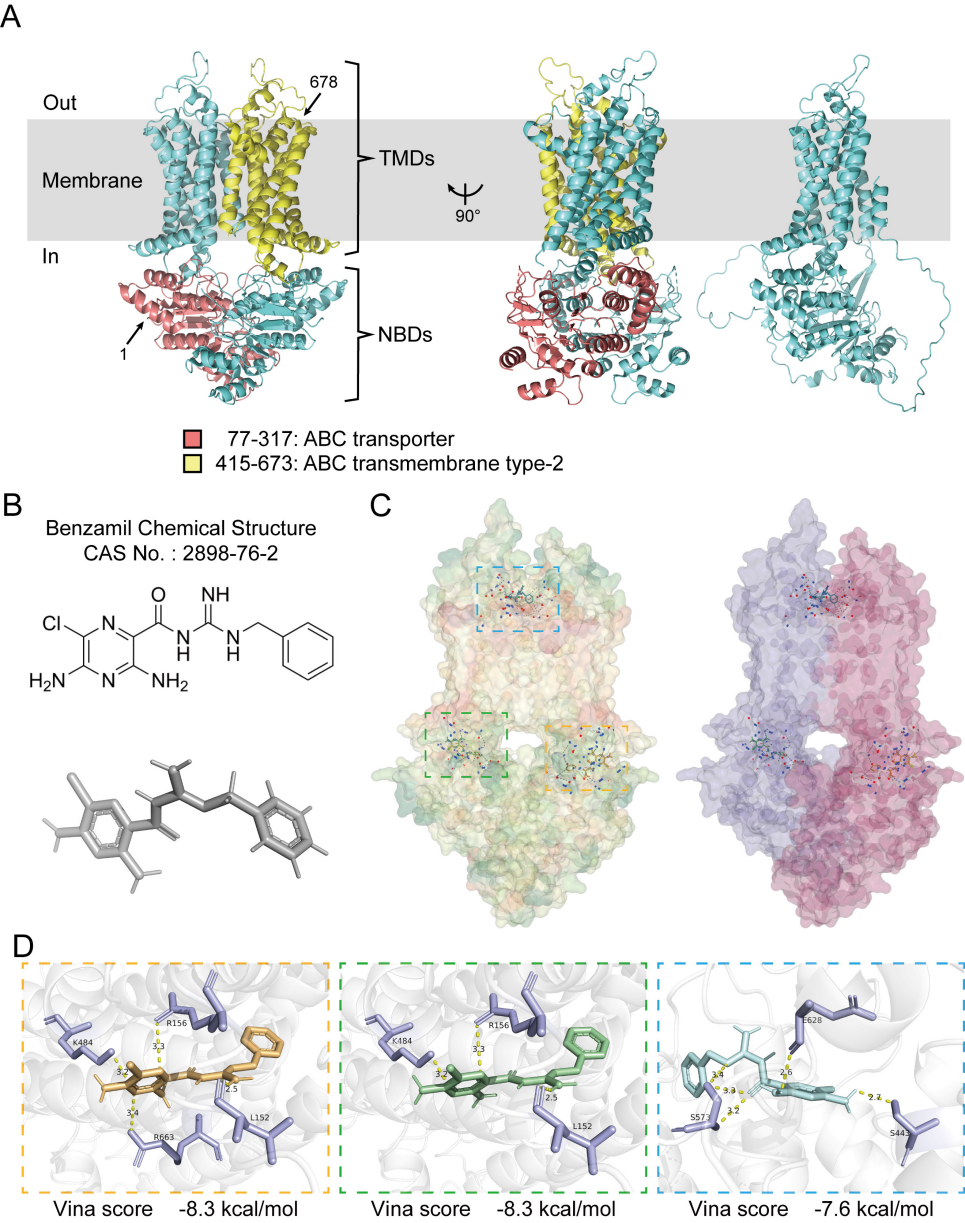
Molecular docking analysis of ABCG1 structure and its inhibitor. **A** The overall structure of ABCG1, with the amino terminus and carboxyl terminus marked by arrows 1 and 678, respectively, the ABC transporter domain in red, the type 2 domain in the ABC transmembrane domain in yellow, and the ABCG1 monomer on the far right. All structural diagrams were plotted in PyMol. **B** Chemical structure and structure prediction of ABCG1 inhibitor Benzamil. **C** Overall structure prediction of ABCG1 and Benzamil molecular docking. ABCG1 structure is shown according to hydrophobicity (left) and chain (right). **D** Docking prediction results show three possible binding sites. The 'Vina score' represents the docking score, the smaller the score, the more stable the docking interaction. We present the corresponding ABCG1 binding sites (in purple) and indicate the contact distances (in yellow).

In an effort to further elucidate the pathways through which ABCG1 influences the progression of ccRCC, we analyzed its structural characteristics and molecular functions. Human ABCG1 is classified as a hemitransporter, comprising a total length of 678 amino acids, which includes an N-terminal nucleotide-binding domain (NBD) and a C-terminal transmembrane domain (TMD), this functional assembly typically exhibits homodimer formation. The ABC transporter domain of ABCG1 encompasses 241 amino acids (residues 77-317), while the transmembrane type-2 domain comprises 259 amino acids (residues 415-673) (Figure 5A).

ABCG1 is predominantly localized to cellular membranes, including the plasma membrane, Golgi membrane, and the endoplasmic reticulum membrane, where it functions to facilitate the efflux of phospholipids (such as sphingomyelin), cholesterol, and its oxygen derivatives (e.g., 7 β-hydroxycholesterol) in an ATP-dependent manner. Benzamil has been identified as an inhibitor of ABCG1 function and belongs to both the pyrazine and guanidine classes of compounds 17-20. Its chemical structure has been predicted and verified (Figure 5B). Molecular docking was performed using CB-Dock2, yielding three results with high binding affinity, which were visualized and exported for further analysis (Figure 5C, D).

**Figure 6:**
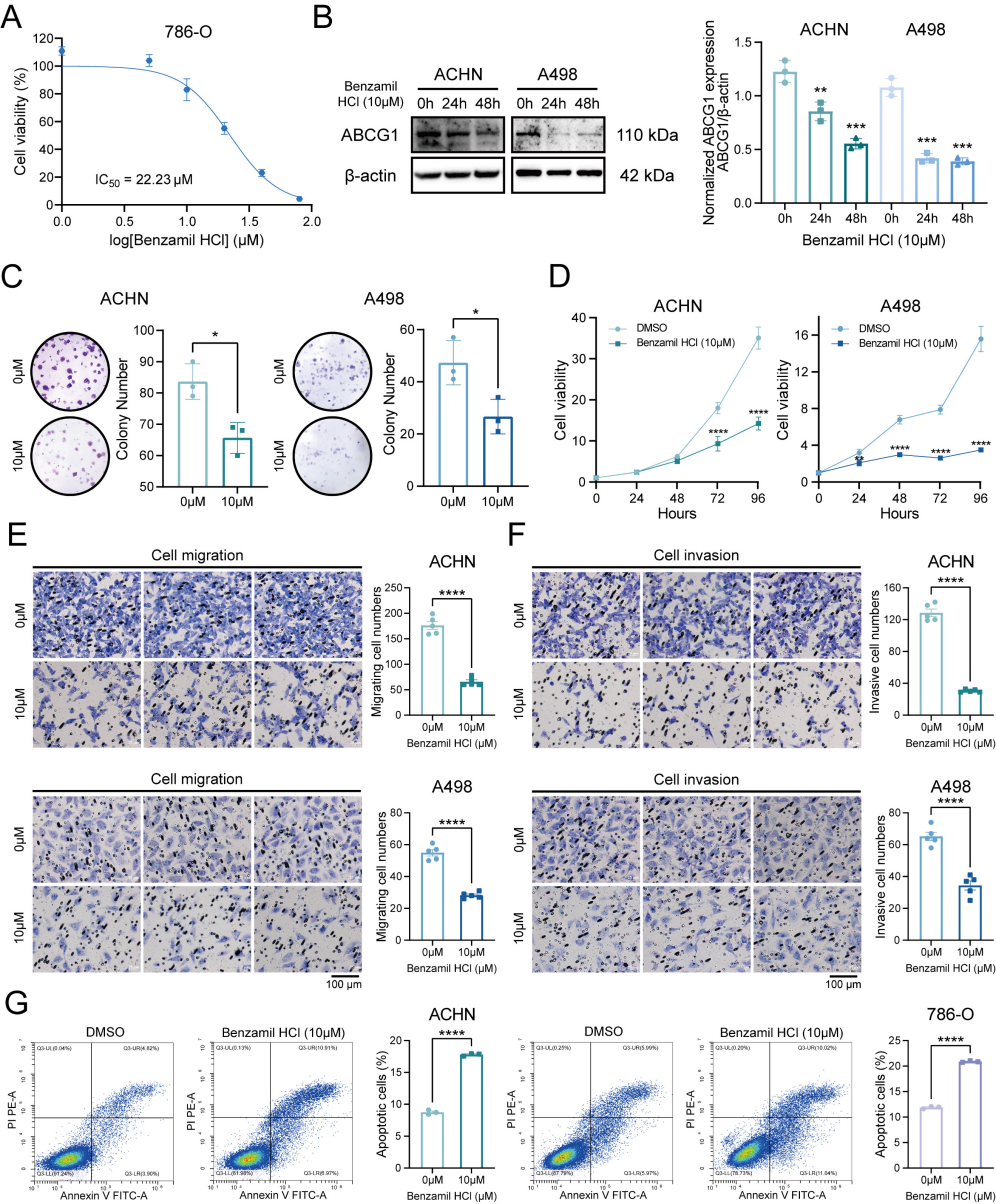
Effects of ABCG1 inhibitors on the function of ccRCC cells. **A** Cytotoxicity assay to detect the IC50 of Benzamil. **B** Western blot to detect the inhibitory efficiency of Benzamil in ACHN and A498 cell lines. Quantitative analysis was performed using Image J software. **C** Plate colony formation assay of ACHN and A498 cell lines. **D** CCK-8 proliferation assay of ACHN and A498 cell lines. **E-F** Migration (**E**) and invasion (**F**) assay of ACHN and A498 cell lines. **G** Flow cytometry to detect the percentage of apoptosis of ACHN and 786-O cell lines using Benzamil. *P < 0.05, **P < 0.01, ***P < 0.001, ****P < 0.0001.

The cytotoxicity assays revealed that the IC_50_ of Benzamil was determined to be 22.23 μM, prompting subsequent experiments to utilize a concentration of 10 μM (Figure 6A). Following the application of the ABCG1 inhibitor, the expression levels of ABCG1 protein in ACHN and A498 cells were found to be inhibited (Figure 6B). Clonogenic and CCK-8 proliferation assays indicated that the proliferation capabilities of ccRCC cells were also diminished following the inhibition of ABCG1 expression (Figure 6C, D). Additionally, transwell assays demonstrated that inhibiting ABCG1 expression reduced both the migratory (Figure 6E) and invasive (Figure 6F) capabilities of ccRCC cells, while levels of apoptosis were found to be elevated (Figure 6G).

### ABCG1 regulates an important pathway in ccRCC progression

**Figure 7:**
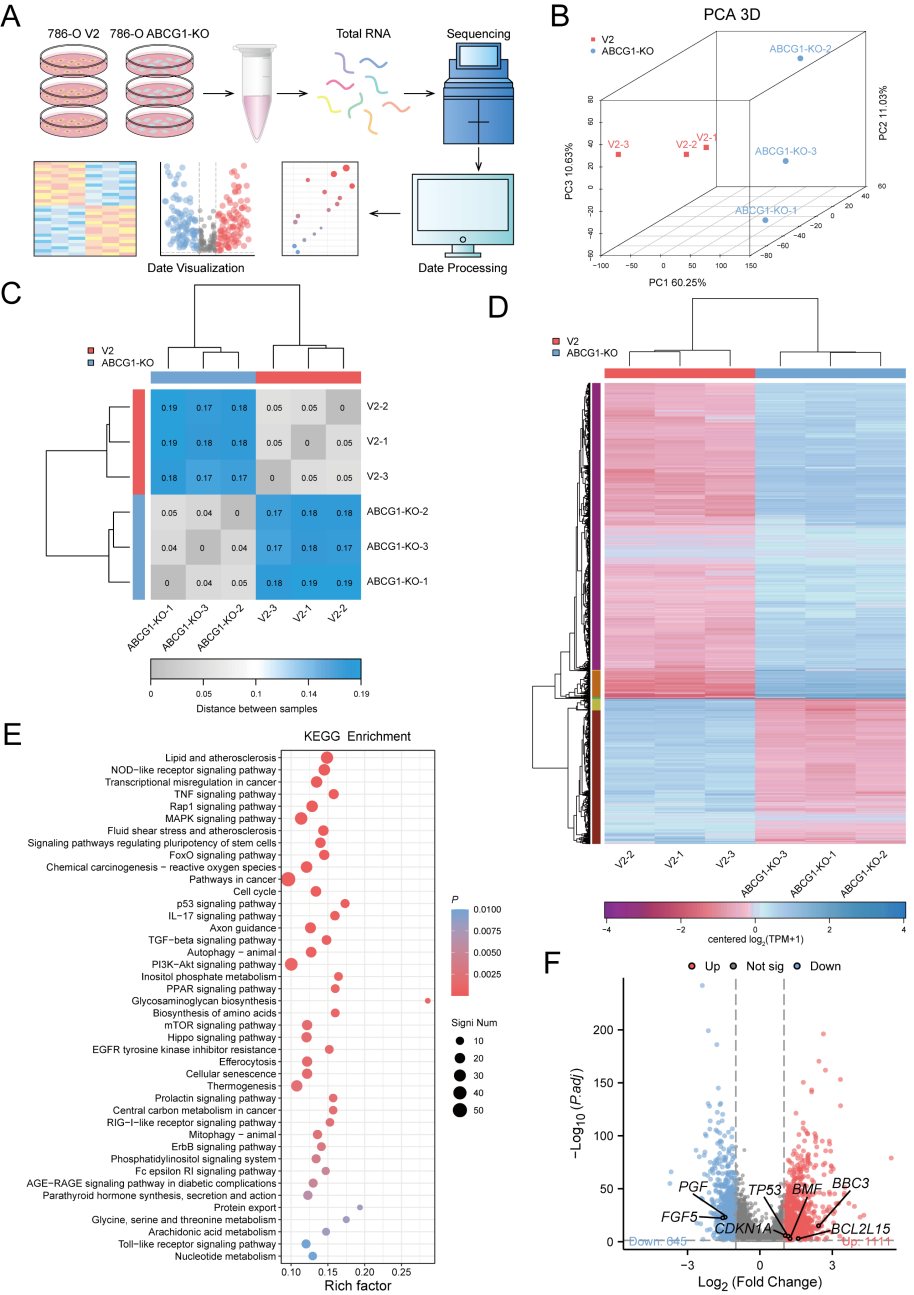
ABCG1 is involved in regulating the progression of ccRCC. **A** RNA-seq flow chart. **B-D** RNA-seq was performed on 786-O cells V2 control group and ABCG1-KO group. (**B**) PCA principal component analysis diagram. (**C**) Heat map of the distance between samples. (**D**) Cluster heat map of DEGs expression. **E** Bubble plot of KEGG enrichment analysis of ABCG1-related DEGs. **F** Volcano plot of the analysis results for ABCG1-related DEGs.

To investigate the role of ABCG1 in regulating the progression of ccRCC, we conducted transcriptome sequencing on the 786-O cell line, comparing the control and ABCG1-KO groups (Figure 7A). Principal component analysis (PCA) was employed to elucidate the differences and relational distances between samples (Figure 7B). A heatmap indicating sample distance provided a visual illustration of sample similarities (Figure 7C). These analyses confirmed the reproducibility of our samples. Subsequent gene cluster analysis of significantly differentially expressed genes resulted in expression pattern visualizations, as presented in heat maps (Figure 7D). KEGG enrichment analysis performed on the filtered DEGs revealed that these genes were enriched in critical pathways associated with lipid metabolism, atherosclerosis, NOD-like receptor signaling, transcriptional dysregulation, p53 signaling, PI3K-AKT signaling, and PPAR signaling, among others, indicating their relevance in the progression of ccRCC (Figure 7E). Considering the molecules involved in apoptosis regulation through *ABCG1* knockout, we identified *TP53*, *CDKN1A*, *BBC3*, *BCL2L15*, *BMF*, and others as relevant candidates. Similarly, the promotion of ccRCC proliferation and migration by ABCG1 was linked to FGF and PGF molecules (Figure 7F).

**Figure 8:**
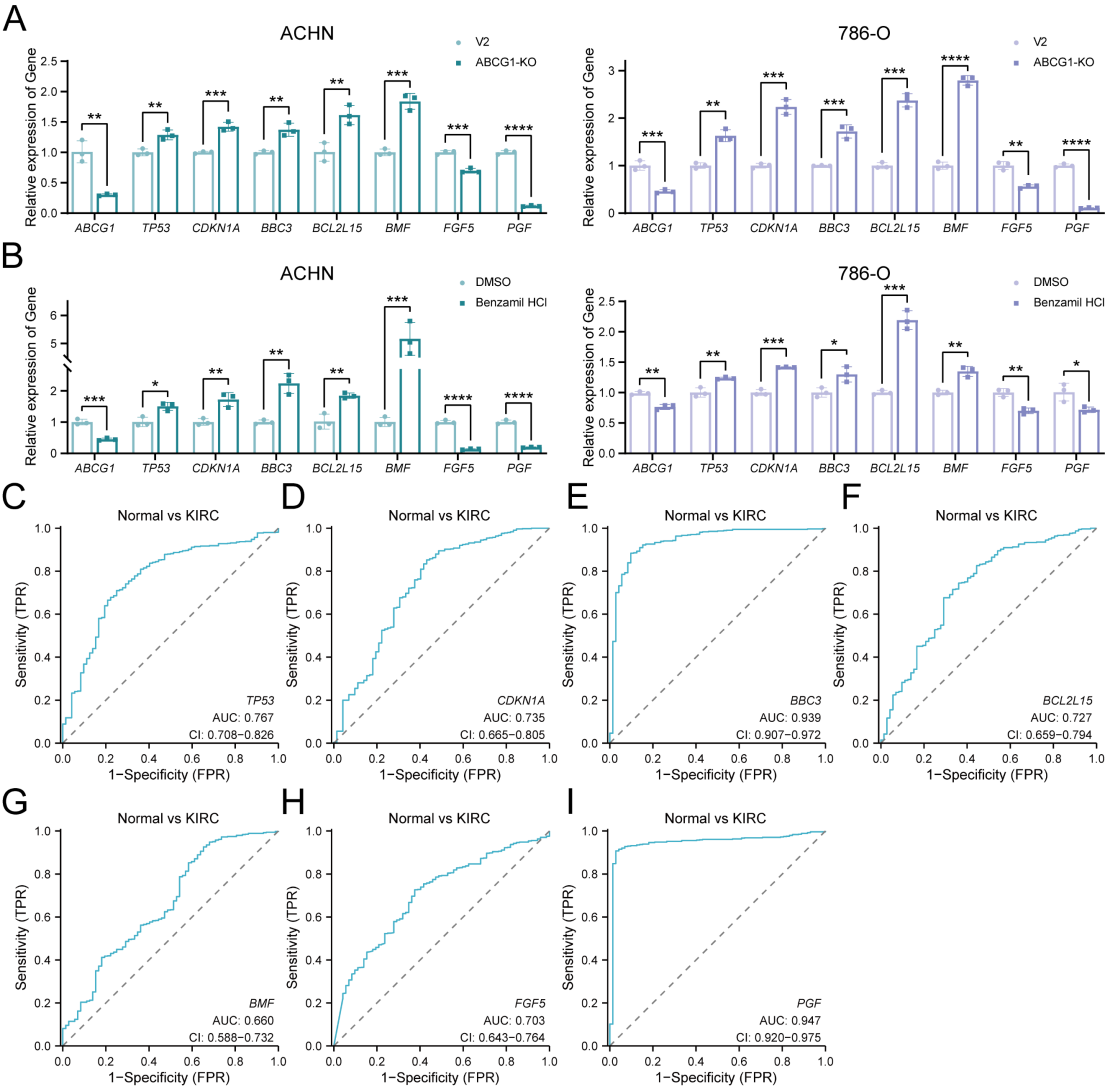
ABCG1 is involved in regulating the apoptosis and proliferation of ccRCC. **A** RT-qPCR was used to detect the changes in the expression levels of apoptosis and proliferation-related differentially impartially in ACHN and 786-O cells in the V2 control group and the ABCG1-KO group. **B** RT-qPCR was used to detect the effect of Benzamil on the expression levels of differentially related genes related to apoptosis and proliferation in ACHN and 786-O cell lines. **C-I** Diagnostic ROC curves of ABCG1-related DEGs in the TCGA-KIRC database. *P < 0.05, **P < 0.01, ***P < 0.001, ****P < 0.0001.

Furthermore, we performed RT-qPCR analyses of apoptosis-related DEGs *TP53*, *CDKN1A*, *BBC3*, *BCL2L15*, *BMF*, alongside proliferation-related DEGs *FGF5* and *PGF*. Our results indicated that ABCG1 exhibited a negative correlation with apoptosis-related genes while positively correlating with proliferation-related genes in both ABCG1-KO cell lines and inhibitor-treated, consistent with our previous phenotypic observations (Figure 8A, B). Analysis through the TCGA database further corroborated that the AUC of these genes surpassed 0.6, signifying their commendable diagnostic value in conjunction with *ABCG1* (Figure 8C-I). In conclusion, these results imply that ABCG1 may influence the expression of various genes and thereby impact the progression of ccRCC.

## Discussion

ABCG1, a member of the adenosine triphosphate binding cassette family, has predominantly been researched within the contexts of atherosclerosis 21, diabetes, and tumor resistance. However, its potential as a tumor biomarker remains under-explored. Existing studies indicate that disruptions in cellular cholesterol homeostasis accompany tumor cell proliferation, with increased serum total cholesterol levels correlating with a reduced cancer risk 22. Moreover, elevated cholesterol concentrations within tumor cells are indicative of heightened cholesterol metabolism in proliferative cancerous tissues 23.

ABCG1 mediates cholesterol efflux via reverse cholesterol transport while also playing a pivotal role in intracellular cholesterol trafficking. Notably, two single nucleotide polymorphisms (SNPs) in ABCG1 have been linked to decreased survival rates among patients with non-small cell lung cancer (NSCLC), highlighting their potential utility as prognostic indicators 24. Furthermore, ABCG1 expression is elevated in prostate adenocarcinoma (PRAD), exhibiting an inverse correlation with overall survival 25. ABCG1 facilitates endoplasmic reticulum (ER) homeostasis and inhibits ER stress-induced apoptosis in low-grade glioma cells, reinforcing its significance for the viability of glioblastoma multiforme (GBM) cells and its status as a potential therapeutic target 26. In aggregate metastatic colon cancer cells, high ABCG1 expression fosters lipid accumulation within extracellular vesicles (EVs), which reduces cytotoxicity and enhances tumor cell survival 27. Contrarily, elevated cytoplasmic ABCG1 levels do not reliably predict outcomes in hepatocellular carcinoma (HCC) patients. However, heightened nuclear ABCG1 expression correlates with poor prognosis, suggesting a critical role for the subcellular distribution of ABCG1 in tumor recurrence 28. The study found that *Abcg1^-/-^* macrophages exhibited an intrinsic bias towards M1 polarization, characterized by increased NF-κB activation and enhanced direct cytotoxicity against tumor cells *in vitro*. These findings highlight the connection between cholesterol homeostasis and cancer 29. Furthermore, the *ABCG1* gene may play a role in cholesterol metabolism, potentially contributing to the pathogenesis and progression of ccRCC. Additionally, ABCG1 has been linked to the innate immune regulation of miR-33, further underscoring its multifaceted roles in tumor biology 30. Collectively, these investigations suggest a potential oncogenic role for ABCG1, yet the mechanisms underpinning its involvement in carcinogenesis and tumor progression warrant additional exploration.

Our study found that ABCG1 is highly expressed in ccRCC patients and is significantly associated with clinical features such as histological stage and M stage. We hypothesize that the decrease in ABCG1 levels during the progression of RCC may suggest that the cholesterol transport-related molecule ABCG1 could be a potential target for ccRCC treatment. Alternatively, it may enhance anti-tumor immunity by activating specific immune responses.

The ROC curve results demonstrated that ABCG1 possesses discernible diagnostic value, while Cox regression analysis indicated that ABCG1 is an independent prognostic factor for ccRCC. These observations suggest that ABCG1 has the potential to emerge as a biomarker for ccRCC. Our study detected ABCG1 in urine, likely carried by extracellular vesicles present in the urine. This finding may provide new insights for the development of non-invasive diagnostic tests for RCC. *In vitro* experiments further elucidated that ABCG1 knockout notably inhibited proliferation, migration, and invasion of tumor cells while concurrently increasing apoptosis levels. Treatment with the ABCG1 inhibitor Benzamil produced similar inhibitory effects, reinforcing our findings. Additionally, RNA sequencing was employed to unravel the mechanisms through which ABCG1 may influence ccRCC progression, revealing enrichment of various classical pathways. Notably, ABCG1 negatively modulated apoptosis-related genes (*TP53*, *CDKN1A*,* BBC3*, *BCL2L15*, *BMF*) while positively regulating proliferation-related genes (*FGF5* and *PGF*). The oncogenic factor *FGF5* is implicated in cell growth, morphogenesis, and tumor invasion, whereas *PGF* is known to enhance cell population proliferation. *TP53* participates in the transcription of long intergenic non-coding RNA p21 (lincRNA-p21) and lincRNA-Mkln1, with lincRNA-p21 facilitating TP53-dependent transcriptional repression leading to increased apoptosis and cell cycle regulation. *CDKN1A* is crucial for cell cycle control and mediates G2 arrest in response to DNA damage 31, while *BBC3* is vital for mediating apoptosis via both TP53-dependent and TP53-independent pathways 32-33. *BCL2L15* is involved in apoptotic processes 33, and *BMF* belongs to the BCL2 family, acting as a regulator of apoptosis. Collectively, these findings suggest that ABCG1 may impact the expression of these genes, thereby modulating the progression of ccRCC.

## Conclusions

ABCG1 is characterized by high expression levels in ccRCC, which may correlate with disease progression. Downregulation of ABCG1 expression impedes the growth of ccRCC cells while promoting apoptosis. ABCG1-associated differentially expressed genes are implicated in various critical pathways governing tumor progression, suggesting a role for ABCG1 in ccRCC progression through its modulation of key proliferation and apoptosis-related gene expressions. Furthermore, both *ABCG1* and these associated genes exhibit considerable diagnostic potential, and their combined analysis may enhance the diagnostic accuracy for ccRCC. The ABCG1 inhibitor Benzamil also proposes a novel approach for the development of molecularly targeted therapies aimed at treating ccRCC. Collectively, these findings position ABCG1 as a promising diagnostic and prognostic biomarker for ccRCC.

## Figures and Tables

**Table 1:** Expression of ABCG1 in RCC patients.

Characteristics		N	ABCG1 expression		*P* value
			-	+	
Gender	Male	42	16	26	0.115
	Female	26	15	11	
Age	< 60	35	12	23	0.347
	≥ 60	33	15	18	
Tumor size	< 4	25	14	13	0.673
	> 4, ≤7	28	14	12	
	> 7	15	6	9	
T stage	T1-2	53	22	31	0.416
	T3-4	15	8	7	
Fuhrman	Ⅰ, Ⅱ	57	22	35	0.325
	Ⅲ, Ⅳ	11	6	5	
Location	Tumor	68	3	65	< 0.0001****
	Para-tumor	68	30	34	

Note: IHC staining results (+): positive, (-): negative. The results were considered statistically significant at *P* < 0.05. ****P < 0.0001.

**Table 2:** Univariate and multivariate analyses of clinicopathologic parameters associated with overall survival.

Characteristics	Total (N)	Univariate analysis		Multivariate analysis	
		Hazard ratio (95% CI)	*P* value	Hazard ratio (95% CI)	*P* value
Age	541				
<= 60	269	Reference		Reference	
> 60	272	1.791 (1.319 - 2.432)	**< 0.001**	1.744 (1.140 - 2.666)	**0.010**
Gender	541				
Male	354	Reference			
Female	187	1.083 (0.796 - 1.473)	0.613		
Pathologic stage	538				
Stage I&Stage II	332	Reference		Reference	
Stage III&Stage IV	206	3.910 (2.852 - 5.360)	**< 0.001**	0.959 (0.373 - 2.469)	0.931
Pathologic T stage	541				
T1&T2	350	Reference		Reference	
T3&T4	191	3.210 (2.373 - 4.342)	**< 0.001**	2.124 (0.925 - 4.877)	0.076
Pathologic N stage	258				
N0	242	Reference		Reference	
N1	16	3.422 (1.817 - 6.446)	**< 0.001**	1.286 (0.633 - 2.611)	0.487
Pathologic M stage	508				
M0	429	Reference		Reference	
M1	79	4.401 (3.226 - 6.002)	**< 0.001**	3.127 (1.836 - 5.328)	**< 0.001**
Histologic grade	533				
G1&G2	250	Reference		Reference	
G3&G4	283	2.665 (1.898 - 3.743)	**< 0.001**	1.464 (0.900 - 2.381)	0.124
*ABCG1*	541				
Low	270	Reference		Reference	
High	271	0.489 (0.358 - 0.667)	**< 0.001**	0.407 (0.259 - 0.639)	**< 0.001**
